# *MET18* Deficiency Increases the Sensitivity of Yeast to Oxidative Stress and Shortens Replicative Lifespan by Inhibiting Catalase Activity

**DOI:** 10.1155/2017/7587395

**Published:** 2017-07-30

**Authors:** Ya-qin Chen, Xin-guang Liu, Wei Zhao, Hongjing Cui, Jie Ruan, Yuan Yuan, Zhiguang Tu

**Affiliations:** ^1^Key Laboratory of Laboratory Medical Diagnostics, Ministry of Education, Chongqing Medical University, No. 1 Yixueyuan Road, Yuzhong District, Chongqing 400016, China; ^2^Institute of Aging Research, Guangdong Medical University, Dongguan 523808, China; ^3^Key Laboratory for Medical Molecular Diagnostics of Guangdong Province, Dongguan 523808, China; ^4^School of Laboratory Medicine, Guangdong Medical University, Dongguan 523808, China

## Abstract

Yeast* MET18*, a subunit of the cytosolic iron-sulfur (Fe/S) protein assembly (CIA) machinery which is responsible for the maturation of Fe/S proteins, has been reported to participate in the oxidative stress response. However, the underlying molecular mechanisms remain unclear. In this study, we constructed a* MET18/met18Δ* heterozygous mutant yeast strain and found that* MET18 *deficiency in yeast cells impaired oxidative stress resistance as evidenced by increased sensitivity to hydrogen peroxide (H_2_O_2_) and cumene hydroperoxide (CHP). Mechanistically, the mRNA levels of catalase A* (CTA1)* and catalase T* (CTT1)* as well as the total catalase activity were significantly reduced in* MET18*-deficient cells. In contrast, overexpression of* CTT1* or* CTA1 *in* MET18*-deficient cells significantly increased the intracellular catalase activity and enhanced the resistance ability against H_2_O_2_ and CHP. In addition,* MET18* deficiency diminished the replicative capacity of yeast cells as evidenced by the shortened replicative lifespan, which can be restored by* CTT1* overexpression, but not by* CTA1*, in the* MET18*-deficient cells. These results suggest that* MET18*, in a catalase-dependent manner, plays an essential role in enhancing the resistance of yeast cells to oxidative stress and increasing the replicative capacity of yeast cells.

## 1. Introduction

Oxygen (O_2_) is crucial for the livelihood of organisms grown aerobically. However, exposure to higher O_2_ concentration can increase the production of reactive oxygen species (ROS), including hydroxyl radical (OH^−^), hydrogen peroxide (H_2_O_2_), and superoxide anions (O^2−^), which may exert detrimental effects on cell growth. ROS can also be generated by redox-cycling chemicals and heavy metals in the environment, as well as by the endogenous metabolic process. In addition, the effects of environmental oxygen concentrations on the intracellular ROS production are highly dependent upon the culture medium, the cells themselves, the metabolic state of the cells, and the presence of any antioxidative compound or enzyme and other known and unknown factors [1–3]. It has been shown that culturing cells in air (21% O_2_) exposes the cells to much higher levels of oxygen than what they are usually submitted to under physiological conditions (2~5% O_2_) [2]. Cells normally have oxidant defense systems to protect themselves against ROS or oxidative stress. However, the underlying mechanisms remain largely unknown [3, 4].

Yeast* MET18*, the homologue of methyl-methanesulfonate sensitivity protein 19* (MMS19)* in human, is a conserved component of the cytosolic iron-sulfur (Fe/S) protein assembly (CIA) machinery in eukaryotes [5–8]. From yeasts to plant cells to human cells, the CIA machinery is generally located in the cytoplasm and responsible for the maturation of Fe/S proteins through catalyzing the synthesis of Fe/S clusters [9, 10]. In the CIA pathway,* MET18* and other subunits, such as* CIA1*,* CIA2*, and nuclear architecture-related protein 1* (NAR1)*, form a CIA complex in which* MET18* functions as a delivering agent by directly interacting with the target protein and transferring the Fe/S cluster to the protein, leading to the maturation of Fe/S proteins [11, 12].* MET18 *could form complexes with Dos2, Rki1, and Cdc20 in order to participate in various cell processes such as DNA repair, RNA polymerase transcription, chromosome segregation, and maintenance of telomere length, among others [13]. Therefore,* MET18* has a number of roles in cell metabolism.

Previous studies have suggested that some components of CIA machinery,* CIA2* and* NAR1*, are associated with yeast response to oxidative stress and thus regulate the replicative lifespan (RLS) of yeast. In* CIA2/cia2Δ* mutants, the deficiency of* CIA2* enhances the sensitivity of yeast to the oxidant cumene hydroperoxide (CHP), resulting in an impaired oxidative stress tolerance and thus a decreased RLS [14, 15]. Similarly,* NAR1*-deficient yeast cells have been found to be more sensitive to O^2−^-generating compound paraquat (PQ) and have shorter RLS than wild-type (WT) yeast strain [16]. Any deficiency in the CIA pathway will impact the activities of enzymes involved in ROS and oxidative stress management [13].

Since* MET18* is also a component of CIA, we hypothesized that* MET18* may play an important role in yeast response to oxidative stress. To confirm this hypothesis, we performed the functional assay for* MET18* mutation by constructing a* MET18/met18Δ *heterozygous mutant yeast strain. Our results demonstrate for the first time that* MET18* deficiency may increase the response sensitivity of yeast to oxidative stress and shorten the RLS through suppressing the expression and activity of catalases.

## 2. Materials and Methods

### 2.1. Yeast Strains and Culture

The yeast strains used in this study are listed in Table 1. All strains were isogenic to BY4743 and were stored in liquid yeast peptone dextrose (YPD; 1% yeast extract, 2% peptone, and 2% glucose; Oxoid, Basingstoke, UK) medium mixed with equal volume of 50% (v/v) glycerol at −80°C. For all experiments, cells were removed from frozen stock and streaked onto solid YPD plate followed by an incubation for 2~3 days at 30°C. Then the single colonies were picked and grown in liquid YPD until the exponential phase using an orbital shaker where yeast cells were shaken at 30°C and 180 rpm.

### 2.2. *MET18/met18Δ* Mutants and Plasmids Construction

The* MET18/met18Δ* mutants were constructed as previously described [14]. Briefly,* URA3* cassette was amplified by polymerase chain reaction (PCR) from pRS306 vector using the following primers: 5′-TGT TTT AAC TGG GAA AAA GCG GAA CAA TTG GGC CTT ACA AGA TTG TAC TGA GAG TGC AC-3′ (forward) and 5′-CGT GCT CAT CAA TGT GAA CAA ATT ATT AAA TAC AAG CGT CTG TGC GGT ATT TCA CAC CG-3′ (reverse) (Invitrogen, Shanghai, China). Then the PCR products were transformed into BY4743 to replace one copy of* MET18* by homologous recombination, and the transformants were selected on SD-URA medium (Clontech, Mountain View, CA, USA). The heterozygous* MET18/met18Δ* cells were verified by PCR using the following primers: 5′-TGT GGC TGT CGT TTC GTG G-3′ (forward) and 5′-TAC AGT TTC CAC TGC GAA CAC A-3′ (reverse) (Invitrogen, Shanghai, China).

The entire coding regions of catalase A* (CTA1)* and catalase T* (CTT1)* were amplified from genomic DNA of BY4743 using primers flanked by* Sac *I and* Xba *I restriction sites (*CTT1* primers: 5′-TAT GTC GAC ATG AAC GTG TTC GGT AAA AAA G-3′ (forward) and 5′-GCG TCT AGA TTA ATT GGC ACT TGC AAT GG-3′ (reverse);* CTA1* primers: 5′-ATA GTC GAC ATG TCG AAA TTG GGA CAA GA-3′ (forward) and 5′-CGC TCT AGA TCA AAA TTT GGA GTT ACT CG-3′ (reverse)) followed by digestion with* Sac *I and* Xba *I (Takara, Otsu, Shiga, Japan) and ligation into pAUR123 vector (Takara, Otsu, Shiga, Japan) to generate pAUR123-*CTT1* and pAUR123-*CTA1 *expressing* CTT1* and* CTA1*, respectively. Subsequently, pAUR123-*CTT1* or pAUR123-*CTA1* was transformed into* MET18/met18Δ *mutants. The empty pAUR123 vector was used as a negative control. The transformants were selected on YPD or SD-URA media containing aureobasidin A (Sigma-Aldrich, St. Louis, MO, USA) and then verified by PCR using the following primers: 5′-TCT GCA CAA TAT TTC AAG C-3′ (forward) and 5′-TTC GTT TTA AAA CCT AAG AGT CAC-3′ (reverse).

### 2.3. Real-Time PCR

Total RNA was isolated from yeast cells using a Yeast RNAiso Kit (Takara, Otsu, Shiga, Japan) followed by cDNA synthesis using a Transcriptor First-Strand cDNA Synthesis Kit with gDNA Eraser (Takara, Otsu, Shiga, Japan). Real-time PCR was performed using SYBR® Premix Ex Taq™ (Takara, Otsu, Shiga, Japan) and the primers are shown in Table 2. Data were normalized to the internal control* PRP8 *[14].

### 2.4. Spot Assay

The sensitivity of yeast to oxidant stressors, including H_2_O_2_, CHP, and PQ (Sigma-Aldrich, St. Louis, MO, USA), was determined by spot assay. Yeast cells were cultured to exponential phase (OD_600_ = 2.0) in liquid YPD followed by a 10-fold dilution with sterile water. Four additional 10-fold serial dilutions were performed, and 5 *μ*L of each dilution was inoculated onto solid YPD plates supplemented with indicated stressors. The plates were incubated at 30°C for 2~5 days for colony formation.

### 2.5. Enzyme Activity Assay and Determination of Intracellular H_2_O_2_ Level

Yeast cells in exponential phase were harvested and washed twice with cold sterile water followed by resuspension in lysis buffer containing acid-washed glass beads and 20 cycles of 10 s vortexing plus 20 s cooling [14]. After centrifugation at 12000 rpm for 15 min, the supernatants were collected and used for an enzyme activity assay and determination of intracellular H_2_O_2_ levels. The protein concentration was determined using the BCA protein Assay Kit (Sangon Biotech, Shanghai, China) following the manufacturer's instruction. The total superoxide dismutase (SOD) activity and the catalase activity were determined using a Total Cellular SOD Activity Kit (Dojindo Molecular Technologies, Rockville, MD, USA) and a Catalase Assay Kit (Beyotime Biotechnology, Shanghai, China), respectively. The intracellular H_2_O_2_ level was determined using an H_2_O_2_ Assay Kit (Beyotime Biotechnology, Shanghai, China).

### 2.6. Replicative Lifespan (RLS) Assay

RLS assay was performed as previously described [15]. Briefly, cells were thawed from frozen stocks and grown on freshly made YPD plate for 2 days and then patched onto the second YPD plate and incubated for an additional 24 h. Cells were then restruck on the third YPD plate and incubated for about 12 h, and then the virgin buds were isolated and used for RLS analysis.

### 2.7. Statistical Analysis

All experiments were repeated at least three times. Data were expressed as the mean ± standard deviation (SD). Statistical significance was assessed by calculating *p* values using a two-tailed Student's *t*-test or Wilcoxon rank-sum test. A value of *p* < 0.05 was considered statistically significant.

## 3. Results

### 3.1. *MET18* Deficiency Enhances the Cellular Response of Yeast to Oxidative Stress

**Figure 1:**
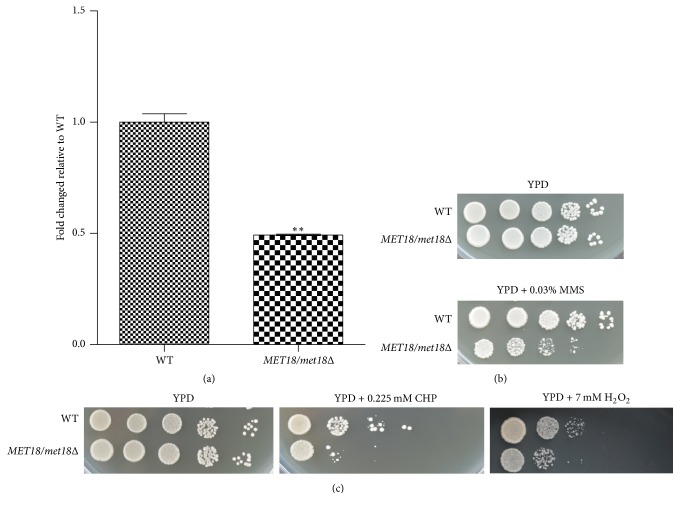
Effects of* MET18* deficiency on yeast response to oxidative stress. (a) mRNA expression of* MET18* under unstressed condition was determined by real-time PCR. (b) Tenfold dilution series of WT and* MET18/met18Δ *cells were spotted on YPD plates with or without 0.03% MMS and then incubated at 30°C for 2~3 days to detect the cellular response to MMS. (c) Tenfold dilution series of WT and* MET18/met18Δ *cells were spotted on YPD plates with 0.225 mM CHP or 7 mM H_2_O_2_ and incubated at 30°C for 2~5 days to detect the cellular response to CHP and H_2_O_2_. Results shown are representative of three independent experiments, ^*∗∗*^*p* < 0.01 versus WT cells (*n* = 3).

To investigate the role of* MET18* in yeast response to oxidative stress, we constructed a* MET18/met18Δ* mutant strain which was confirmed by a real-time PCR detection of* MET18*- and* URA3*-specific PCR products. As shown in Figure 1(a), the mRNA level of* MET18* was decreased by more than 50% in* MET18-*deficient cells compared with WT cells, indicating that the expression of* MET18* mRNA in* MET18/met18Δ *mutants was successfully suppressed by the replacement of one copy of* MET18* with* URA3* gene.

We then sought to examine the cellular response of* MET18/met18Δ *mutants to oxidative stressors, including H_2_O_2_ and CHP. A DNA-damaging agent methyl-methanesulfonate (MMS) was used as a positive control [17]. As shown in Figures 1(b) and 1(c), the significant decreases in yeast growth in response to MMS, H_2_O_2_, and CHP were observed in* MET18/met18Δ *mutants, compared with those in WT cells, suggesting that* MET18* deficiency may increase the oxidative stress response in yeast cells.

### 3.2. *MET18* Deficiency Decreases Catalase Activity While Increasing Intracellular H_2_O_2_ Levels

**Figure 2:**
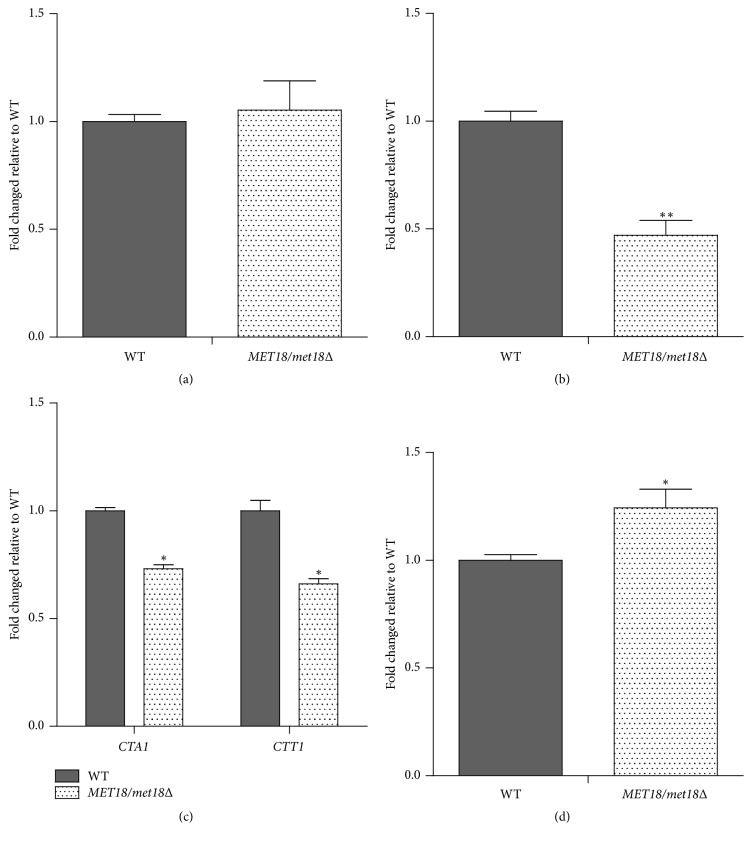
Effects of* MET18* deficiency on enzymatic defense system against oxidative stress. Total SOD activity (a), catalase activity (b),* CTA1* and* CTT1* mRNA levels (c), and intracellular H_2_O_2_ levels (d) under unstressed condition were determined and compared between WT and* MET18/met18Δ *cells. Results shown are representative of three independent experiments. ^*∗*^*p* < 0.05, ^*∗∗*^*p* < 0.01 versus WT cells (*n* = 3).

To further examine whether* MET18* is involved in enzymatic antioxidant defense against oxidative stress, we determined the activities of SOD and catalases which are responsible for ROS scavenging [18, 19]. As shown in Figures 2(a) and 2(b), the activity of total SOD in* MET18/met18Δ *mutants was comparable to that in WT strain, whereas the total catalase activity was significantly decreased by more than 50% in* MET18*-deficient cells compared with WT strain. Meantime, the mRNA expression of both* CTT1* and* CTA1 *was significantly downregulated in* MET18*-deficient cells (Figure 2(c)). Conversely, H_2_O_2_ levels in* MET18/met18Δ *mutants were higher than those in WT strain (Figure 2(d)), suggesting an increased oxidative stress in* MET18/met18Δ *mutants. These results indicate that* MET18* deficiency may downregulate antioxidant defense enzymes, leading to the increased oxidative stress.

### 3.3. Either* CTT1* or* CTA1* Restores the* MET18* Deficiency-Suppressed Oxidative Stress Resistance in Yeast

**Figure 3:**
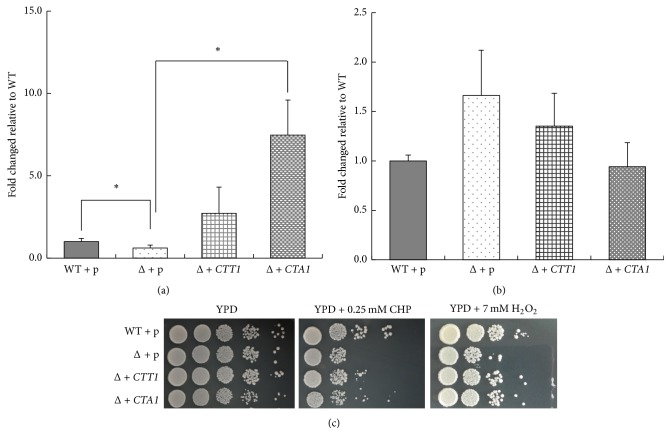
Effects of* CTT1* or* CTA1* on oxidative stress resistance in* MET18/met18Δ* cells. Catalase activity (a) and the intracellular H_2_O_2_ levels (b) under unstressed conditions were determined and compared among pAUR123-transfected WT strain (WT + p), pAUR123-, and* CTT1*- and* CTA1*-transfected* MET18/met18*Δ mutants ((Δ + p), (Δ +* CTT1*), and (Δ +* CTA1*), resp.). ^*∗*^*p* < 0.05 versus WT cells (*n* = 3). (c) Tenfold dilution series of WT + p, Δ + p, Δ +* CTT1*, or Δ +* CTA1* were spotted on YPD plates with or without the stressors, as indicated, and incubated at 30°C for 2~3 days to detect the cellular response. Results shown are representative of three independent experiments.

To investigate if the increased oxidative stress response in* MET18/met18Δ* mutants is attributable to inhibition of catalases, pAUR123-*CTT1* or pAUR123-*CTA1* was transfected into* MET18/met18Δ* mutants to generate* CTT1*- or* CTA1*-overexpressing mutants. As shown in Figure 3(a), the catalase activity in* MET18/met18Δ* mutants overexpressing* CTT1 *(*Δ *+* CTT1*) or* CTA1 *(*Δ *+* CTA1*) was significantly increased as compared with the empty vector-transfected WT strain (WT + p) or the empty vector-transfected mutant strain (*Δ *+ p). Furthermore,* CTT1*- or* CTA1*-overexpression also reduced the intracellular levels of H_2_O_2_ (Figure 3(b)), consistent with our findings (Figure 2). Importantly,* CTT1*- or* CTA1*-overexpression greatly enhanced the resistance of* MET18/met18Δ* mutants against H_2_O_2_ and CHP (Figure 3(c)), leading to an increased cell growth. In contrast,* CTT1*- or* CTA1*-overexpression did not appear to promote cell growth under unstressed conditions (Figure 3(c)), suggesting the important role of catalases in oxidative stress response of* MET18/met18Δ* mutants. These results indicate that inhibition of* CTT1 *or* CTA1 *activity may be a major mechanism underlying the impaired oxidative response in* MET18*-deficient yeasts.

### 3.4. *CTT1*, but Not* CTA1*, Restores the Lifespan of* MET18*-Deficient Yeast

**Figure 4:**
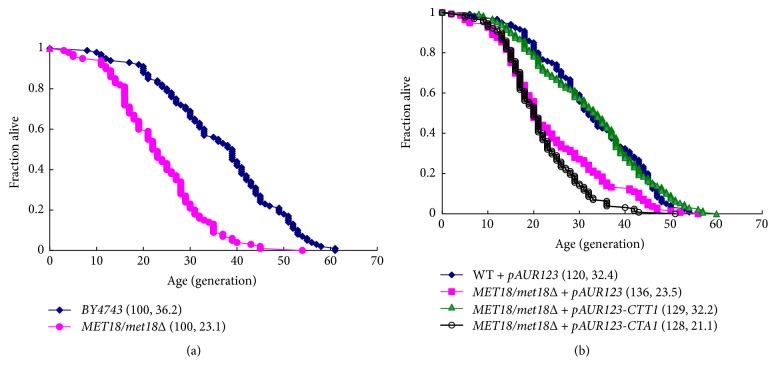
Effect of* CTT1* or* CTA1* on RLS in* MET18/met18Δ *mutants. (a) RLS of WT and* MET18/met18Δ* cells; (b) RLS of WT and* MET18/met18Δ *cells transfected with pAUR123,* CTT1, *or* CTA1*. Results shown are representative of three independent experiments.

To further examine if* MET18* has effects on yeast aging, we performed a RLS assay in WT strains and* MET18/met18Δ* mutants. The results showed that* MET18/met18Δ* mutants had a shortened RLS compared with the WT strain BY4743 (mean lifespan: 23.1 versus 36.2) (Figure 4(a)). Of note, overexpression of* CTT1* rescued* MET18* deficiency-induced RLS defects, whereas overexpression of* CTA1* did not appear to affect RLS in* MET18/met18Δ* mutants (Figure 4(b)), suggesting that* MET18* deficiency leads to shortened RLS or aging in yeast, at least in part, through inhibition of catalases.

## 4. Discussion

In the present study, we constructed a* MET18/met18Δ* mutant using the WT BY4743 yeast strain and examined the response of* MET18*-deficient yeast to oxidative stress caused by H_2_O_2_ and CHP. Our results suggest that* MET18* deficiency increases the cellular response of yeast to H_2_O_2_ and CHP and shortens RLS through downregulation of catalases* CTT1* and* CTA1*.

It has been reported that* MET18* participates in oxidative stress responses [20, 21]; however, the underlying mechanism remains unknown. Oxidative stress is termed as a disruption of cellular redox balance due to the failure of oxidant defense system to scavenge excess ROS which can be generated from normal cellular metabolism in living organisms and is a potential damage to cells [22]. In our study, the total catalase activity as well as the mRNA levels of both* CTT1* and* CTA1 *was significantly decreased in* MET18/met18Δ* mutant compared with the WT strain (Figures 2(b) and 2(c)), resulting in an increased level of intracellular H_2_O_2_ in mutant cells (Figure 2(d)). These results indicate that the cellular redox balance is abolished in* MET18*-deficient cells due to the decreased antioxidants (catalase) and increased ROS (H_2_O_2_) levels.

Since* MET18* is a component of the CIA machinery and plays an important role in the maturation of Fe/S proteins [5, 6], one possible explanation for the downregulation of catalase is that the Fe/S cluster transfer required for catalase maturation is inhibited in* MET18*-deficient yeast, resulting in H_2_O_2_ accumulation and an increase in the oxidant burden of the cells. In addition,* MET18* could be responsible for or has a role to play in Fe/S cluster transfer or epigenetic modifications in yeasts, affecting their metabolism [7, 13, 23]. Indeed, Wang et al. [13] showed that* MET18* can regulate active DNA methylation and methylation pathways in* Arabidopsis*. Buzas et al. [23] showed that* AtDRE2*, a member of the CIA, had epigenetic functions that are independent of the CIA. Duan et al. [7] showed that* MET18*-dependent transfer of the iron cluster was required for* ROS1*-mediated active DNA methylation. Nevertheless, additional studies will be necessary to specify the role of* MET18* in oxidative stress of the yeasts.

H_2_O_2_, at high concentrations, primarily contributes to oxidative damage [20, 21]. Dismutation of O^2−^ by SOD is a major source of H_2_O_2_ [24]. However, in our study, the activity of SOD in* MET18/met18Δ *mutants did not appear to be different from that in WT strain (Figure 2(a)), suggesting that other enzymes or factors than SOD are responsible for the increased H_2_O_2_ level in* MET18*-deficient cells. Catalases may downregulate the intracellular level of H_2_O_2_ by catalyzing the decomposition of H_2_O_2_ into water and oxygen [25]. Accordingly, the increased H_2_O_2_ level and enhanced response to oxidative stress have been observed in yeast cells lacking* CTA1* and* CTT1* [24, 25], which is consistent with our findings. It seems that, compared with WT strains,* MET18/met18Δ* mutants are more sensitive to the oxidative stress caused by H_2_O_2_ and CHP due to the decreased catalase activity and the subsequent H_2_O_2_ accumulation.

There are conflicting findings in regard to the role of catalases in yeast response to H_2_O_2_. Some studies show that the activity of* CTT1 *is essential to protect yeast cells against H_2_O_2_ challenge, whereas the activity of* CTA1* is dispensable [25]. On the other hand, some studies indicate that both* CTT1* and* CTA1 *mutants are sensitive to H_2_O_2_, suggesting that* CTT1* and* CTA1 *are equally important for antioxidative response of yeast to H_2_O_2_ stress [26]. Our study reveals that the overexpression of either* CTT1* or* CTA1* in* MET18/met18Δ* mutants can effectively scavenge the accumulated intracellular H_2_O_2_ and thus enhances the cellular resistance to CHP and H_2_O_2_ (Figure 3).

Catalase activity is also closely associated with lifespan regulation in various species. It has been reported that overexpression of human catalase targeted to mitochondria can extend mouse lifespan by eliminating mitochondrial H_2_O_2_ [2]. Consistently, reduced mitochondrial ROS as well as increased antioxidant enzyme activities of SOD, catalase, and glutathione peroxidase 1 has been observed in long-lived white-footed mice compared with common laboratory mice [27]. The role of catalase in the yeast replicative aging may be complicated by genetic background (haploid or diploid) and nutrient availability (glucose or ethanol) [28]. Several lines of evidence show that catalases and excess H_2_O_2_ exert positive and negative effects on RLS in yeast, respectively [29]. Our study found that loss of one copy of* MET18* leads to the reduction in yeast RLS which can be rescued by* CTT1*, but not by* CTA1* (Figure 4), indicating that inactivity of* CTT1* is a driving force of senescence in* MET18/met18Δ* mutants. These findings are in agreement with the previous study that* CTT1*, but not* CTA1*, is required for the maintenance of RLS in yeast cultured in glucose-containing media [30].* MET18* deficiency leads to shortened RLS or aging in yeast, at least in part, through inhibition of catalases. It is known that* CTT1* and* CTA1* are not submitted to the same regulatory mechanisms in response to nutrient availability and oxidative stress [30]. This could explain, at least in part, why* CTT1* and* CTA1* did not have the same effect on RLS in the present study, but additional studies are necessary to address this issue. Nevertheless, Rona et al. [2] showed that* CTT1* overexpression increased the lifespan of calorie-restricted* S. cerevisiae* deficient in* Sod1* through increased peroxide degradation catalyzed by catalase. Similarly, Zhao et al. [31] showed that* CTT1* overexpression increased the replicative lifespan of* S. cerevisiae* sensitive to MMS and deficient in* KSP1*. On the other hand, Ohtsuka et al. [32] showed that* CTT1* was required for the H_2_O_2_-resistant phenotype, but that* CTT1* overexpression did not increase the chronological lifespan of the yeast. These previous studies provide some clues about the role of catalases as modulators of replicative lifespan in* S. cerevisiae*.

## 5. Conclusions

In conclusion, our study demonstrates for the first time that* MET18 *deficiency in yeast impairs oxidative stress tolerance and shortens lifespan, at least partly, through the inhibition of catalase activity and accumulation of intracellular H_2_O_2_. Further investigation is needed to examine the role of* MET18* in other Fe/S transfer-associated processes such as epigenetic modifications in eukaryotes.

## Figures and Tables

**Table 1:** Yeast strains in this study.

Strain name	Genotype	Comments	Source
BY4743	*MATa/MATα*his3Δ1/his3Δ1 leu2Δ0/leu2Δ0 lys2/lys2Δ0 met15Δ0/met15 ura3Δ0/ura3Δ0	Wild-type strain	Gift from Matt Kaeberlein
GDMUB1001	BY4743 *MET18::URA3*	Deletion of one copy of *MET18* in BY4743	This experiment
GDMUB1002	BY4743 + pAUR123	BY4743 harboring pAUR123	This experiment
GDMUB1003	BY4743*MET18::URA3 + *pAUR123	GDMUB1001 harboring pAUR123	This experiment
GDMUB1004	BY4743*MET18::URA3 + *pAUR123*-CTA1*	GDMUB1001 harboring pAUR123*-CTA1*	This experiment
GDMUB1005	BY4743*MET18::URA3 + *pAUR123*-CTT1*	GDMUB1001 harboring pAUR123*-CTT1*	This experiment

**Table 2:** The real-time PCR primers used in this study.

Gene	Primers	Sequence
*PRP8*	Forward	TCATGGCTGCGTCTGAAGTA
	Reverse	GGCTCAAACCCTTCCGATAG
*MET18*	Forward	TGCTGGAAGTTGTCGTTGC
	Reverse	TCGTTTTTGGAGAGGTGGTC
*CTT1*	Forward	GATTCCGTTCTACAAGCCAGAC
	Reverse	GGAGTATGGACATCCCAAGTTTC
*CTA1*	Forward	CCAACAGGACAGACCCATTC
	Reverse	TTACCCAAAACGCGGTAGAG
